# Hyponatremia complicating labour—rare or unrecognised? A prospective observational study

**DOI:** 10.1111/j.1471-0528.2008.02063.x

**Published:** 2009-01-28

**Authors:** V Moen, L Brudin, M Rundgren, L Irestedt

**Affiliations:** a Department of Anaesthesiology and Intensive Care, County HospitalKalmar, Sweden; b Section of Anaesthesiology and Intensive Care, Department of Physiology and Pharmacology, Karolinska InstituteStockholm, Sweden; c Department of Medicine and Health Sciences, University Hospital LinköpingLinköping, Sweden; d Department of Physiology and Pharmacology, Karolinska InstituteStockholm, Sweden

**Keywords:** Epidural, fluids, hyponatraemia, labour, obstetric, oxytocin

## Abstract

**Objective:**

The aim of this study was to investigate the occurrence of hyponatraemia following delivery, with a hypothesis that hyponatraemia has a high prevalence in labouring women.

**Design:**

Prospective observational study.

**Setting:**

Consultant-led delivery suite in County Hospital, Kalmar, Sweden.

**Sample:**

A total of 287 pregnant women at term (37 full gestational weeks).

**Methods:**

Oral fluids were allowed during labour. Blood samples were collected on admission, after delivery, and from the umbilical artery and vein.

**Main outcome measure:**

Hyponatraemia defined as plasma sodium ≤130 mmol/l after delivery.

**Results:**

Hyponatraemia was found in 16 (26%) of the 61 mothers who received more than 2500 ml of fluid during labour. Two-thirds of fluids were orally ingested. Decrease in plasma sodium concentration during labour correlated with duration of labour and the total fluid volume administered. Analysis by multivariate logistic regression showed that hyponatraemia was significantly correlated with fluid volume (*P* < 0.001) but not with oxytocin administration or epidural analgesia. Hyponatraemia correlated significantly with prolonged second stage of labour, instrumental delivery, and emergency caesarean section for failure to progress (*P* = 0.002).

**Conclusions:**

Hyponatraemia is not uncommon following labour. Tolerance to a water load is diminished during labour; therefore, even moderate fluid volumes may cause hyponatraemia. Women should not be encouraged to drink excessively during labour. Oral fluids, when permitted, should be recorded, and intravenous administration of hypotonic fluids should be avoided. When abundant drinking is unrecognised or intravenous fluid administration liberal, life-threatening hyponatraemia may develop. The possibility that hyponatraemia may influence uterine contractility merits further investigation.

## Introduction

The risks and dangers of maternal hyponatraemia during labour have previously been recognised.^1^–^3^ Oxytocin administration in glucose solutions has often been claimed to cause hyponatraemia. Maternal hyponatraemia can also be reflected in the fetus, and some studies have reported increased incidence of respiratory distress and hyperbilirubinaemia in hyponatraemic infants.^4^,^5^ In recognition of these studies, changes of obstetric practice were proposed, including administration of oxytocin in sodium-containing fluids.^1^,^6^ Many delivery suites restrict oral intake during labour as a preventive measure, believed to reduce the risk of pulmonary aspiration should general anaesthesia become necessary. The question of optimal fluid administration has been addressed in studies indicating that higher volumes of intravenous fluid during labour have positive effects on the progress of labour.^7^,^8^ No conclusive results have been published regarding energy requirements during labour.^9^ In absence of scientific evidence, advice to pregnant women is based on beliefs and poorly founded recommendations. The habit of drinking large quantities of water has become quite common in the general population, and pregnant women are often advised that larger quantities are needed in pregnancy and during labour.^10^ Abundant drinking during labour has been reported to cause severe and symptomatic hyponatraemia in mothers and infants.^11^–^14^ We conducted a prospective observational study to investigate the hypothesis that the reported cases of hyponatraemia were not merely isolated cases but represented the extremes in a population with high prevalence of hyponatraemia.

## Materials and methods

### Patients and study protocol

The study was conducted from January until June 2007 in the Department of Gynaecology and Obstetrics at Kalmar County Hospital, Sweden. The consultant-led delivery suite has approximately 1400 deliveries annually. After approval by the local ethical committee, women were informed about the study during antenatal classes. All women at term (37 full gestational weeks) were eligible with patient refusal as the only exclusion criterion. During the study period, 554 eligible women were delivered, of these 46 declined study participation, and 213 women were not included as they were uninformed or very near delivery. To complete the control group of women delivered by elective caesarean section, an additional 13 women were enrolled before study completion in September 2007. After written informed consent, 308 women were included in the study, but 21 women were later excluded as blood sample analysis were incomplete, leaving 287 participants in the study groups (125 nulliparas and 162 multiparas).

During labour, women were allowed to drink freely, but no solids were permitted, a policy commonly adopted in Sweden.

Blood samples were collected from the mothers on admission and as soon as possible following delivery. Whenever possible, an extra blood sample was taken before emergency caesarean section. During caesarean section, maternal blood samples were collected immediately following delivery and also after 24 hours. Clamped cord blood samples from umbilical artery and vein were collected. Maternal and umbilical cord blood samples were analysed with i-STAT 1 Analyzer (Celite Corporation, Santa Barbara, CA, USA). Plasma osmolality in maternal blood samples was analysed by the method of cryoscopy (Advanced Osmometer Model 3D3; Molek AB, Enskede, Sweden). Postnatal data from infants affected by significant congenital malformation or disease, meconium aspiration, or sepsis would be excluded from analysis.

Oxytocin for augmentation of labour was administered in 5% glucose at a concentration of 20 mU/ml. Intravenous glucose could also be ordered by the obstetrician as caloric supplement. Ringer's acetate (500–1000 ml) was administered intravenously during epidural analgesia. Ringer's acetate and ephedrine were administered intravenously for blood pressure control during caesarean section, all performed under regional anaesthesia. The volume of Ringer's acetate administered perioperatively during caesarean section was estimated to be 600 ml before delivery. This fluid volume is not included in the volumes administered before delivery.

### Statistics

Women were recruited to permit the inclusion of at least 30 women in each of four groups composed according to duration of labour and obstetric outcome. In addition, 30 women delivered by planned caesarean section were included as controls. A mean decrease of the sodium concentration of 5 mmol/l during labour would be considered significant. The sample size of at least 26 women in each group was required for a 90% power to detect a significant difference with a two-sided alpha error of 0.05 (Table 1). Mothers or infants with missing data were excluded from the corresponding analysis only. Group differences were analysed using nonparametric tests for continuous parameters (Mann–Whitney *U* test when comparing two groups and Kruskal–Wallis analysis of variance when more than two groups were compared). Spearman's rank correlation was used for association between two variables. Categorical variables were compared with chi-square test if the number of subjects was appropriate, otherwise Fisher's exact test was used. Initial analysis of the results indicated that major clarity of the presentation could be achieved by reallocating the study participants to three new groups composed according to total fluid administration during labour (Table 2).

**Table 1:** Baseline values and observed changes related to fluids given until birth. Group differences analysed using Kruskal–Wallis nonparametric ANOVA and Mann–Whitney *U* test

	Control group elective caesarean (*n* = 26)		Total fluids given until birth						Kruskal–Wallis *P* value	Mann–Whitney *P* value
					
			Fluid group 1 <1000 ml (*n* = 113)		Fluid group 2 1000–2500 ml (*n* = 87)		Fluid group 3 >2500 ml (*n* = 61)			
						
	Median	Q1–Q3	Median	Q1–Q3	Median	Q1–Q3	Median	Q1–Q3		
**Mother**										
Age	32	27–35	31	28–34	30	27–33	30	26–33	0.117	
Weight before pregnancy (kg)	67	58–78	66	59–73	67	60–77	66	60–77	0.750	
Body mass index before pregnancy	26.2	22.6–28.1	23.7	21.0–25.5	24.1	21.5–27.9	23.9	22.1–27.1	0.066	
Weight at term (kg)	83	72–89	79	71–89	80	74–90	83	74–91	0.399	
Weight increase	12	10–18	13	11–16	14	10–17	15	11–19	0.466	
Gestational weeks	38.5	38.3–38.7	39.7	39.0–40.6	40.0	39.1–40.9	40.3	39.6–41.0	0.087	
Cervical dilatation at admission (cm)	—	—	5.0	4.0–7.0	4.0	3.0–4.0	3.0	2.0–4.0	<0.001	<0.001
Duration of labour (hours)	—	—	1.6	0.9–3.0	5.9	4.3–8.9	12.2	9.8–14.7	<0.001	<0.001
Duration second stage (hours)	—	—	0.2	0.1–0.5	0.5	0.2–1.0	0.6	0.4–1.0	<0.001	<0.001
Oral fluids (ml/hour)	0	0–0	185	79–316	196	136–305	196	154–263	0.429	
Intravenous fluid (ml/hour)	Not measured	Not measured	0	0–0	52	3–102	94	53–144	<0.001	<0.001
Total fluid (ml/hour)	Not measured	Not measured	194	100–325	291	201–362	314	260–360	<0.001	<0.001
Total fluid (ml)	Not measured	Not measured	400	200–575	1680	1355–2050	3570	2925–4355	—	
Oxytocin (ml)	—	—	0	0–0	50	0–166	200	100–400	<0.001	<0.001
Oxytocin (units)	—	—	0.0	0.0–0.0	1.0	0.0–3.3	4.0	2.0–8.0	<0.001	<0.001
Oxytocin (maximum rate mU/minute)*	—	—	10	5–20	20	10–30	40	20–60	<0.001	<0.001
Duration epidural (hours)	—	—	1.7	1.7–1.7	4.3	2.9–6.1	8.8	6.8–10.6	—	
Osmolality at baseline (mOsm/kg)	279	277–281	280	277–282	280	276–282	279	277–281	0.501	
Osmolality postpartum (mOsm/kg)	280	277–282	282	279–284	277	276–281	274	272–277	<0.001	<0.001**
Na at baseline (mmol/l)	137	136–138	137	136–137	136	135–137	136	135–138	0.238	
Na postpartum (mmol/l)	137	137–138	136	135–137	135	133–136	133	130–135	<0.001	<0.001**
Na change (mmol/l)	0.0	−1.0 to 1.0	0.0	−1.0 to 1.0	−2.0	−3.0 to −1.0	−3.0	−5.0 to −1.0	<0.001	<0.001**
Glucose at baseline (mmol/l)	5.0	4.5–5.5	5.1	4.7–5.8	5.2	4.9–5.9	5.3	4.8–6.2	0.133	
Glucose postpartum (mmol/l)	4.8	4.2–5.1	6.7	5.9–7.9	7.7	6.5–9.4	7.8	6.9–9.4	<0.001	<0.001**
**Umbilical cord**										
Arterial BE	−2.0	−3.0 to −1.0	−4.0	−6.0 to −3.0	−5.0	−7.0 to −3.0	−6.0	−9.0 to −3.0	<0.001	0.006**
Arterial pH	7.27	7.24–7.29	7.26	7.22–7.31	7.24	7.19–7.29	7.24	7.17–7.29	0.042	0.014***
Arterial Na (mmol/l)	139	138–140	139	137–140	137	136–139	136	134–138	<0.001	<0.001**
Arterial–venous Na difference (mmol/l)	1.0	0.0–1.0	1.0	0.0–2.0	1.0	0.0–2.0	1.0	0.0–2.0	0.736	
Arterial glucose (mmol/l)	3.4	3.0–3.7	4.2	3.6–5.0	5.2	4.2–6.1	5.4	4.7–6.6	<0.001	<0.001**
**Infant**										
Birthweight (g)	3415	3120–3790	3535	3305–3850	3670	3360–4160	3805	3580–4040	0.001	0.001**

All group differences are compared with Kruskal–Wallis nonparametric analysis of variance (ANOVA). When significant differences are found, Mann–Whitney *U* test is performed to analyse differences between fluid groups 1 and 3.

* Including only subjects who received oxytocin. Maximum rate mU/minute: maximum rate of oxytocin infusion during first stage of labour.

** Differences between control group elective caesarean and fluid group 3: *P* < 0.001 (Mann–Whitney) (additional significant test).

*** Difference between control group elective caesarean and fluid group 3: *P* = 0.14 (Mann–Whitney) (additional significant test).

**Table 2:** Baseline values and total fluids (oral plus intravenous) given until birth

	Control group elective caesarean (*n* = 26) *n*(%)	Total fluids given until birth			Chi-square *P* value	Fischer *P* value
				
		Fluid group 1 <1000 ml (*n* = 113) *n*(%)	Fluid group 2 1000–2500 ml (*n* = 87) *n*(%)	Fluid group 3 >2500 ml (*n* = 61) *n*(%)		
Initial groups*						
Vaginal <4 hours	—	88 (78)	20 (23)	1 (2)		
Vaginal >4 hours, no epidural	—	13 (12)	34 (39)	6 (10)		
Vaginal >4 hours with epidural	—	0 (0)	30 (34)	40 (66)		
Emergency caesarean section	—	12 (11)	3 (3)	14 (23)		
Elective caesarean section	26 (100)	0 (0)	0 (0)	0 (0)		
**Parity**						
Nullipara	12 (46)	23 (20)	44 (51)	46 (75)		
Multipara	14 (54)	90 (80)	43 (49)	15 (25)	<0.001	<0.001
**Complications**						
Gestational diabetes	0 (0)	0 (0)	3 (3)	0 (0)		
Pre-eclampsia	0 (0)	3 (3)	0 (0)	5 (8)		
Hypertension	0 (0)	5 (4)	1 (1)	1 (2)		
**Onset of labour**						
None	26 (100)	5 (4)	0 (0)	0 (0)		
Induction	—	8 (7)	7 (8)	8 (13)		
Spontaneous	—	100 (88)	80 (92)	53 (87)	0.455**	
**Delivery**						
Vaginal, spontaneous	—	98 (87)	71 (82)	37 (61)	0.001***	0.001
Vaginal, instrumental***	—	3 (3)	13 (15)	10 (16)		
Caesarean section	26 (100)	12 (11)	3 (3)	14 (23)		
**Indication for caesarean section**						
Breech	17 (65)	6 (5)	0 (0)	0 (0)		
Previous uterine operation	7 (27)	0 (0)	0 (0)	0 (0)		
Fetal asphyxia	—	1 (1)	1 (1)	1 (2)		
Failure to progress	—	2 (2)	2 (2)	12 (20)		
Others	2 (8)	3 (3)	0 (0)	1 (2)		
**Analgesia**						
Epidural****	—	1 (1)	36 (41)	52 (85)		
Spinal	—	1 (1)	3 (3)	0 (0)		
**Oxytocin**						
Yes	—	14 (12)	57 (66)	59 (97)	<0.001	<0.001
≥5 units	—	1 (1)	12 (14)	27 (44)		0.012*****
**Maternal p-Na**						
≤130 mmol/l	0 (0)	1 (1)	4 (5)	16 (26)	<0.001	0.001
**Maternal p-glucose**						
>12 mmol/l	0 (0)	2 (2)	2 (2)	7 (11)	0.013	0.009
**Infant**						
Apgar <7 at 1 minute	0 (0)	4 (4)	5 (6)	5 (8)	0.219	
Apgar <7 at 5 minutes	0 (0)	1 (1)	0 (0)	0 (0)		
Weight loss >10%	1 (4)	2 (2)	3 (3)	5 (8)		0.050
Hypoglycaemia	2 (8)	4 (4)	3 (3)	2 (3)		1.00
Respiratory problem	1 (4)	5 (4)	7 (8)	8 (13)	0.065	

Group differences are compared with chi-square test. When significant differences are found or when cases are few, Fischer's exact test is performed to analyse differences between fluid groups 1 and 3.

* Vaginal <4 hours or <4 hours: Vaginal delivery, labour of shorter or longer duration than 4 hours. Epidural: epidural or spinal analgesia.

** Induction versus spontaneous onset of labour.

*** Vaginal spontaneous delivery versus instrumental vaginal delivery plus delivery by emergency caesarean section.

**** One woman received combined spinal-epidural analgesia during labour.

***** Calculation only includes women who received oxytocin for augmentation of labour.

Univariate and multivariate logistic regressions were performed to study the relationship between maternal hyponatraemia and parity, age, and body mass index as well as fluid volumes administered, epidural analgesia, and oxytocin during labour. All tests were two tailed, and a *P* value of <0.05 was considered statistically significant. The software used was Statistica release 7.1 (Statistica; StatSoft®, Tulsa, OK, USA)

## Results

**Figure 1:**
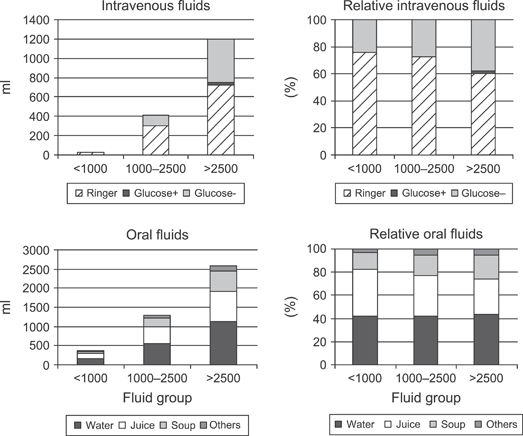
Oral and intravenous fluids during labour in the three fluid groups (mean values used). Mean sodium content of intravenous fluids was 69 mmol/l. In addition to the 130 women who received oxytocin intravenously, 93 women received Ringer's acetate during neuraxial analgesia, and 22 women received caloric supplement as glucose 50 or 100 mg/ml. Sport drinks are included among oral fluids called ‘others’. Ringer, Ringer's acetate; Glucose+, Glucose 50 or 100 mg/ml with Na 50 mmol/l; Glucose−, Glucose 50 or 100 mg/ml without electrolytes.

**Figure 2:**
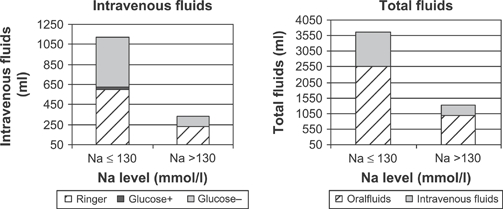
Fluids related to maternal hyponatraemia ≤130 mmol/l (mean values used). Hourly oral fluid intake was similar in all women (*P* = 0.65), but hourly intravenous infusion rates were higher in hyponatraemic women (*P* < 0.001). However, the resulting hourly fluid volumes were similar in all women (*P* = 0.46). Glucose was administered intravenously as energy supply to 22 women, of these 8 women developed hyponatraemia ≤130 mmol/l after having received a mean of 600 ml glucose intravenously (range 100–1500 ml). The 14 women who did not develop hyponatraemia received a mean of 664 ml (100–2000 ml) There was no significant difference between the groups (*P* = 0.8). Ringer, Ringer's acetate; Glucose+, Glucose 50 or 100 mg/ml with Na 50 mmol/l; Glucose−, Glucose 50 or 100 mg/ml without electrolytes.

**Figure 3:**
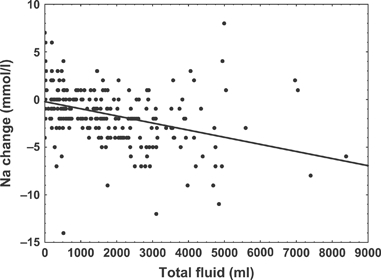
Variation of maternal plasma sodium (Na change) during labour and caesarean section and total fluid volumes administered. Reduction in plasma sodium is significantly correlated with total fluid volume (*P* < 0.001).

Baseline values regarding age, weight, plasma sodium, and plasma osmolality were similar in all groups (Table 1). Roughly two-thirds of all fluids were administered orally, and the remaining one-third was administered intravenously (Table 1 and Figure 1). Twenty-one women (15 nulliparous and 6 multiparous) developed hyponatraemia defined as plasma sodium ≤130 mmol/l during delivery (Table 2 and Figure 2). Plasma glucose above12 mmol/l was found in six of these hyponatraemic women. Reduction in plasma sodium was significantly correlated with the duration of labour and with total fluid volume administered during labour (Figure 3). Analysis by multivariate logistic regression showed that maternal hyponatraemia was significantly correlated with total fluid volume administered during labour, but not with epidural analgesia, or oxytocin administration (Table 3). The lowest maternal plasma sodium after delivery was 122 mmol/l, and the corresponding plasma sodium in the umbilical artery was 126 mmol/l. No participant developed signs of severe hyponatraemic encephalopathy. Maternal reduction in plasma sodium correlated with longer duration of second stage (Spearman's rank correlation, *n* = 218, *r* = 0.35, *P* < 0.001). Maternal reduction in plasma sodium was also significantly larger following instrumental vaginal delivery and emergency caesarean section for failure to progress compared with reduction in plasma sodium following spontaneous vaginal delivery (Mann–Whitney *U* test, *n* = 42, *n* = 202, *P* = 0.002). In women delivered by emergency caesarean section, plasma sodium even tended to be lower before caesarean section than immediately after delivery. However, this difference was not statistically significant (*P* = 0.2). No woman developed hyponatraemia following caesarean section. Umbilical arterial sodium concentration showed significant correlation with postpartum maternal values (*P* < 0.001) but was higher than maternal levels in all groups.

**Table 3:** Interrelationships between maternal hyponatraemia (≤130 mmol/l) immediately postpartum and the various variables analysed with univariate (left) and multivariate (right) logistic regressions

Parameter	Total	Na ≤130	Na ≤130 (%)	Univariate logistic regression		Multivariate logistic regression*	
					
				OR (95% CI)	*P* value	OR (95% Cl)	*P* value
Fluid group**							
<1000	113	1	0.9	1.00		1.00	
1000–2500	87	4	4.6	6.8 (2.9–15.7)		5.2 (2.2–12.7)	
>2500	61	16	26.2	46 (8.5–247)	<0.001	27 (4.6–162)	<0.001
Oxytocin							
<5 units	221	10	4.5	1.00		1.00	
≥5 units	40	11	27.5	8.0 (3.1–21)	<0.001	2.6 (0.9–7.4)	0.072
Epidural							
No	172	3	1.7	1.00			
Yes	89	18	20.2	14.3 (4.1–50)	<0.001	—	—
Parity							
Multipara	148	6	4.1	1.00			
Nullipara	113	15	13.3	3.6 (1.4–9.7)	0.011	—	—

Body mass index and age are not shown (*P* = 0.62 and *P* = 0.59, respectively, in the univariate analysis and are therefore not included in the multivariate analysis).

* Parity was deleted from the multivariate analyses with *P* = 0.86 and epidural with *P* = 0.12.

** Fluid groups as defined in Tables 1 and 2. The control group elective caesarean section is not included in the analysis.

## Discussion

### Principal findings and interpretation

Hyponatraemia defined as plasma sodium ≤130 mmol/l was found in 21 women after delivery. The reduction in plasma osmolality confirms the dilutional origin of hyponatraemia. Hyperglycaemia might have contributed to hyponatraemia in the six hyperglycaemic women.^15^ All study participants drank similar amounts per hour and received moderate hourly volumes of intravenous fluids with a mean sodium content of 69 mmol/l. Intravenous fluids were thus hypotonic, but with a sodium content higher than the most common glucose solutions. Two-thirds of all fluids were orally ingested, and these fluids are invariably hypotonic. In women with longer duration of labour, the cumulative effect of approximately 300 ml of hourly fluid intake resulted in a significant reduction of plasma sodium levels. The duration of labour itself cannot explain the development of hyponatraemia. On the contrary, longer lasting labour without administration of fluids would result in dehydration and hypernatraemia. Hyponatraemia may possibly have a negative influence on the process of labour, as hyponatraemia was significantly correlated with longer duration of second stage, instrumental delivery, and emergency caesarean for failure to progress. In women delivered by emergency caesarean section, hyponatraemia was not caused by intraoperative fluids as this was present before initiating anaesthesia. Earlier studies analysed cord sodium concentrations and found no difference in maternal and cord sodium concentration.^1^,^3^ We analysed both umbilical arterial and venous sodium concentrations and found that umbilical arterial concentrations were higher than maternal levels but significantly correlated, indicating fetal equilibration with maternal hyponatraemia. In infants, large weight loss, hyperbilirubinaemia, and respiratory distress have been believed to be caused by fluid overload.^4^,^5^,^12^ In our study, weight loss in excess of 10% of birthweight was most frequent in fluid group 3 (*P* = 0.05), indicating a possible relationship with maternal fluid overload. The few cases of hyperbilirubinaemia and respiratory problems were evenly distributed, with no differences between the groups (Table 2).

### Hyponatraemia in the obstetric patient

Sodium and the corresponding anions are the main osmoles in extracellular fluid, and sodium concentration mainly reflects body water content. Reabsorption of water in the collecting ducts of the kidney is controlled by vasopressin, in turn regulated by plasma osmolality.^15^ Vasopressin is secreted in response to increased osmolality, causing water retention that will restore osmolality. Reduction of plasma osmolality by approximately 10 mOsm/kg occurs in early pregnancy mainly as a result of a decrease in plasma sodium by 3–5 mmol/l. By 12 weeks of gestation, this adaptation has stabilised, and plasma osmolality remains low until after delivery. Release of vasopressin in response to adequate physiological stimuli corresponds to the nonpregnant state, but a lower osmolality is maintained.^16^ The maximum capacity of excreting a water load at rest is reported as approximately 900 ml per hour in healthy women but is reduced by one-third in late pregnancy.^17^ Pain, stress, and fear are nonosmotic stimuli for vasopressin secretion. Hypovolaemia is, however, the most potent stimulus for vasopressin secretion and may cause water retention even in the presence of hypoosmolality.^18^ Oxytocin, a short peptide with a structure similar to vasopressin, may also cause water retention by stimulation of the specific V2 vasopressin receptors in the kidney. This receptor is not downregulated during prolonged stimulation as is the case with the oxytocin receptor. However, the infusion rate of oxytocin must exceed 20 mU/minute to cause antidiuresis in humans.^19^ Hyponatraemia during labour caused by oxytocin administered in electrolyte-free solutions is well recognised, but despite this, hypotonic solutions are often used as a vehicle for oxytocin.^20^ In our study, fluid volumes needed to administer oxytocin were modest, whereas remaining fluid administration was unrestricted (Table 1). Multivariate logistic regression showed that total fluid volume correlated significantly with hyponatraemia (*P* < 0.001), whereas oxytocin administration did not significantly correlate with hyponatraemia (*P* =0.072) (Table 3). Women in fluid groups 2 and 3 received oxytocin at rates necessary for oxytocin to express antidiuretic effect but probably during too short a period of time for significant antidiuretic effect to develop. The administration of hypotonic fluids in conditions with increased vasopressin activity can cause dilutional hyponatraemia.^15^,^18^,^21^ Typically, this may occur postoperatively, but during labour stimuli for vasopressin release are abundant. Labour itself is, however, not a situation causing inappropriate vasopressin secretion.^22^ The women in our study received fluid volumes well below their predicted maximum capacity of renal excretion at rest; therefore, the development of hyponatraemia indicates increased vasopressin activity during labour. Our results indicate that fluid volume is the major determinant of hyponatraemia, but the antidiuretic effects of endogenous vasopressin and oxytocin administration increase the susceptibility of women to develop hyponatraemia during labour. Fetal vasopressin levels are high during birth, thus also exposing the overhydrated fetus to the risks of hyponatraemia.^12^

Hyponatraemia is usually defined as a decrease in plasma sodium level below 136 mmol/l.^15^ More important than the absolute level of hyponatraemia is the speed with which it has developed. Hyponatraemia causes oedema and cellular swelling; initial symptoms of cerebral oedema are irritability, headache, nausea, and vomiting. Subsequently, convulsions and coma can occur, and severe hyponatraemic encephalopathy can cause respiratory arrest and death.^15^,^21^ Diagnostic difficulties during labour are obvious as initial symptoms of hyponatraemic encephalopathy are nonspecific and may easily be confused with symptoms of pre-eclampsia.

In the pregnant woman, symptoms might possibly occur at a lower level of plasma sodium due to the pregnancy-induced reduction in plasma sodium. Images by computed tomography show reversible reduction in brain size during pregnancy.^23^ Although the significance of this reduction remains to be explained, it could be a result of intracellular adaptation to the pregnancy-induced reduction of extracellular osmolality.

The highest morbidity and mortality rates in hyponatraemic encephalopathy are found in women of fertile age.^18^,^21^ When life-threatening symptoms occur, therapy must aim at quick restitution of plasma osmolality. Some authors advocate even the use of hypertonic saline and diuretics.^15^ In all other cases, the simple measure of water depletion will allow plasma levels to return to normal.

### Fluids during labour

Practice and policies regarding oral intake during labour show large variations throughout the world. A liberal attitude seems to prevail in the UK, many European countries, and Australia, and some countries even allow solids during labour.^9^ Clear oral fluids are recommended during uncomplicated labour in the USA.^24^‘Nil per mouth’ policies are often challenged, particularly by midwives, and create the need of intravenous fluid administration. Maternal starvation may cause fetal acidosis, and several studies address the safety of glucose administration.^25^,^26^ Solutions containing glucose are usually hypotonic despite electrolyte addition, although near isotonic solutions have been used for study purposes.^26^

Two studies indicate that women receiving 250 ml per hour of Ringer's lactated solution intravenously had shorter duration of labour and less need for oxytocin than those receiving 125 ml per hour.^7^,^8^ The assumption regarding beneficial influence of larger volumes of fluids during labour is largely based on literature in the field of sports medicine.^7^,^8^,^27^ For optimal muscle performance, athletes are recommended to drink ‘the maximal amount tolerated’ as the sensation of thirst is believed to underestimate the real fluid requirements during exercise.^27^ Thirst is, however, a strong physiologic stimulus, effectively protecting against dehydration when fluids are readily available. No physiologic warning system protects the body against over-hydration. Therefore, suppression of thirst by abundant drinking implies exposure to the risk of hyponatraemia. Since 1991, several deaths due to hyponatraemic encephalopathy have occurred during endurance competitions. Common recognition of excessive drinking as the main cause of these disasters was, however, delayed until 1995.^28^,^29^

The duration of labour equals a marathon for many women, hence the importance of careful administration of fluids. Many authors consider 150–200 ml per hour safe to drink during labour, but with simultaneous intravenous administration, this could well be an excessive amount as illustrated by the present study.^1^,^12^,^13^ Also, the tonicity of fluids determines their potential for causing hyponatraemia, and clear oral fluids are invariably hypotonic. Sport drinks are somewhat confusingly described as isotonic. However, their osmolality, even when similar to that of plasma, is largely made up of carbohydrates. Their content in sodium is less than half compared with plasma, rendering these drinks hypotonic. Some prospective randomised trials have been designed to study the impact of sport drinks and carbohydrate intake on labour duration and outcome.^30^–^33^ These studies show conflicting results, but all have in common the lack of control of electrolyte status in the study participants.

### Strengths and limitations of the study

A study published in 1981 described iatrogenic hyponatraemia to be caused mainly by electrolyte-free intravenous fluids administered as vehicle for oxytocin.^1^ The authors suggested that oxytocin should be administered at higher concentrations, and fluid balance supervised closely during labour.

The first advice changed practice of oxytocin administration, but more recent case reports of hyponatraemia during labour imply that the second advice was forgotten.^34^,^35^ Hyponatraemia during labour, now less frequently iatrogenic, is more often caused by overdrinking.^12^–^14^,^31^ Our study was therefore designed to evaluate the prevalence of hyponatraemia during modern management of labour. The women in our study received oxytocin in moderate fluid volumes, and most of the fluids administered were orally ingested. Nonetheless, 21 women in our study developed hyponatraemia during labour, thus illustrating that the oral route of administration does not diminish the risks of excess water. As in other studies, we found hyponatraemia in women receiving oxytocin as well as epidural analgesia.^1^,^6^ The multivariate regression analysis performed in our study shows that hyponatraemia was probably caused by fluid intake, but only associated with oxytocin and epidural analgesia. Modern low-dose epidural regimens do usually not call for large volumes of intravenous fluids to preserve blood pressure.

Our results are all the more important as the habit of drinking large quantities of water has become quite common in the general population.^10^ Also, a more liberal attitude to fluid administration during labour might have developed. The comparison of fluid volumes administered during labour as reported in scientific papers indicates that such could be the case. In a study published in 1991, evaluating saline or glucose as vehicle for oxytocin, one study group received a mean of 710 ± 640 ml of intravenous glucose.^2^ A more recent work, published in 2005, studied the effect of unrestricted oral carbohydrate intake.^32^ The intervention group received 3234 ± 1473 ml of intravenous fluids.

The observational study design has limitations when compared with a randomised controlled trial. However, it can be doubted whether an ethical committee would permit a deliberate exposure to the degree and risks of hyponatraemia observed in the present study. Analysis would have benefited from hourly registration of fluid intake and urine output. This option was considered, but although desirable, the work load imposed could have jeopardised the realisation of the study. Also, such close monitoring of behaviour would have introduced an observational bias. However, it is possible that the women who developed hyponatraemia had larger hourly oral fluid intake leading to symptomatic hyponatraemia. Tiredness and irritability, initial symptoms of hyponatraemia, may have prompted energy supplement by intravenous glucose infusion. This alterative interpretation strengthens the need of registering all oral fluid intakes during labour and remembering hyponatraemia as possible diagnosis. Not all consecutive women were included, omitting many women in advanced labour. The proportion of nulliparas is therefore large in our study population, probably increasing the incidence of hyponatraemia. Infants were examined and treated according to departmental routine; therefore, only clinically significant symptoms were investigated. The results regarding neonatal outcome should be interpreted with these limitations in mind.

## Conclusions

Our results indicate that current policies of fluid administration during labour may cause significant hyponatraemia in a large proportion of women. Hyponatraemia during labour has been recognised and studied many decades ago; it is therefore surprising that hyponatraemia during labour still occurs.^29^,^30^

One reason might be that studies in obstetric literature indicate that higher fluid volumes improve labour outcome.^7^,^8^ In addition, midwives focus their attention on caloric requirements believed to be greatly increased during labour, and consequently, encourage oral or intravenous energy supplies.^9^ Moreover, women are informed through media that high volumes of fluids are essential to preserve health.^10^ Tolerance to a water load is, however, diminished during labour; therefore, even moderate fluid volumes may cause hyponatraemia, as experienced by several women in our study.^17^

The highly significant correlation of hyponatraemia with prolonged duration of second stage and instrumental delivery could indicate a causal relationship, but the influence of hyponatraemia on uterine contractility has not been studied. Our results advocate further studies in this area.

Although no severe symptoms of hyponatraemic encephalopathy occurred in any mother or infant in our study, the high prevalence of hyponatraemia imply that hyponatraemic encephalopathy could occur in the larger population of labouring women and their offspring.

We suggest that oral fluid intake during labour should be recorded, and intravenous administration of hypotonic fluids be avoided. The policy of liberal fluid administration should be questioned. Obstetricians and midwives should recognise that hyponatraemia during labour is not uncommon, potentially harmful but easily avoidable, and inform pregnant women accordingly.

### Disclosure of interests

None declared.

### Contribution to authorship

V.M. planned the study with assistance from L.I., L.B., and M.R. V.M. conducted the study. L.B. analysed the data. All authors wrote the paper.

### Details of ethics approval

The study was approved by the ethical committee at the University of Linköping, Sweden (M91-06). All study participants signed informed consent before being enrolled in the study.

### Funding

The study was supported by a grant from Kalmar County Research and Development Committee. The study was planned and performed, the results analysed, and the article written without any involvement of the funders.
